# A Comparison of Generic and Subject‐Specific Finite Element Models of Distal Femur Fractures Treated With Locking Plates

**DOI:** 10.1002/cnm.70043

**Published:** 2025-05-19

**Authors:** Gareth Buhl, Pankaj Pankaj

**Affiliations:** ^1^ Institute for Bioengineering School of Engineering, The University of Edinburgh Edinburgh UK

**Keywords:** bone strain, CT scans, finite element analysis, fracture healing, fracture treatment, Interfragmentary motion, material property mapping

## Abstract

While the need for employing subject‐specific computational biomechanics models for treatment planning in orthopaedics is being increasingly voiced, it has not been clear when such specificity is essential and for which questions simpler models might be adequate. This study uses a novel modelling approach to generate finite element models to examine the influence of subject‐specificity in the treatment of distal femur fractures. Three subject‐specific finite element models are created from clinical CT scans, and the proposed approach is employed to impose identical fractures and locking plate treatments upon them. Additionally, the performance of the generic two‐material model based on a Sawbones fourth generation femur is also evaluated. Interfragmentary motions, plate stresses, and strains at the screw‐bone interface are examined due to a physiological loading at different stages of healing. The study finds that subject‐specificity has a major effect on strains in the bone at the screw‐bone interface. However, interfragmentary motions at the far cortex and plate stresses show minimal sensitivity to subject‐specific factors, while near‐cortical and shear interfragmentary motions are influenced by them. The influence of subject‐specificity decreases as healing progresses. These results indicate that while generic approaches may be sufficient to calculate global assembly responses, material heterogeneity and subject‐specific bone stock variations have a large impact on the interaction between the screws and bone. The study also shows that the proposed method, which enables manipulating bone geometry while retaining subject‐specific properties, can be used to evaluate the influence of subject‐specificity for other orthopaedic simulations.

## Graphical abstract

This study examines the influence of subject‐specific parameters on locking‐plate treated distal femur fractures. A new approach is developed to impose identical injuries and treatments on subject‐specific models. The study finds that plate stress and far cortical interfragmentary motion are not influenced by subject−specificity, while the strain state in the bone at screw insertion sites is highly sensitive. These results, and the new modelling approach used, will help to improve the assessment of future treatments.

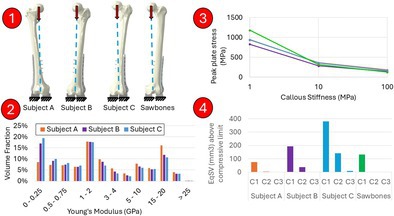

## 1 Introduction

Distal femur fractures are a serious and debilitating category of orthopaedic trauma characterized by a complete fracture of the lower, or distal, part of the femur. These injuries account for 29% of all non‐hip femoral fractures [1] and have been shown to have high rates of complication. This may be because these fractures are particularly difficult to treat due to the complex geometry of the distal femur, the variation in bone density in the region, and the proximity to the delicate articular structures in the knee joint. Even when initial surgery is deemed successful, up to 18% of treatments can result in non‐union [2]. As such, Distal Femoral Fracture (DFF) has been called an “unsolved problem” in the field of orthopaedic surgery [3].

There are a number of treatment options for distal femoral fractures: plates, intramedullary nails, external fixations, and prosthesis. Amongst these, the most commonly employed are intramedullary nails and locking plates. The use of locking plates is particularly favoured in osteoporotic distal femoral fractures, considering their proprieties in resisting varus collapse and having multiple points of fixation [4, 5]. For extra‐articular distal femur fractures, an increasingly common treatment option is the lateral locking plate [6]. These plates stabilize the fracture by affixing to the bone fragments on the lateral side and are intended to provide adequate stiffness and strength to allow the patient to regain function quickly [7]. The performance of locking plates has been studied extensively using clinical reviews [8], mechanical tests [9], and numerical models [4, 10]. Output variables typically measured in plate assembly assessment include peak plate stress, which helps predict mechanical plate failure due to overloading [11] or fatigue [12, 13]; interfragmentary strains (IFS), which have been shown to influence tissue differentiation and healing in the fracture gap [14]; and local bone strains surrounding screw insertion points, which are linked to screw loosening [15, 16].

When conducting mechanical testing or developing numerical models to study the mechanics of this fixation technique, models with generic geometry and material properties are often used rather than considering subject‐specific variations. In some fields of orthopaedic biomechanics, the inclusion of subject‐specific bone density distributions in finite element (FE) models has been impactful and widely adopted; for example, in estimating the risk of femoral neck fracture in osteoporotic patients [17]. However, this technique has only rarely been used in the analysis of plated fracture fixations [18], and never to compare inter‐subject variation in bio‐mechanical response to distal femur fracture fixation. In other words, the question as to how important subject‐specificity is in the treatment simulation of distal femur fractures has not been previously addressed.

The standard approach for creating patient‐specific computer models is to use scans of a patient in need of treatment [19]. As fractures in different patients are dissimilar, comparison cannot be readily made. Another possible approach is to use scans of different healthy subjects, computationally induce similar fractures, and use simulation to examine how a treatment affects biomechanical response in different subjects. This approach has not been previously considered. A possible reason is that most current methods of generating subject‐specific FE models from CT scan data result in the creation of an orphan mesh, in which the material properties are implicitly tied to FE elements comprising the geometry. This means that any geometric alterations, such as those required to introduce screw‐holes and a fracture gap, cannot be readily applied after CT image segmentation and meshing. As these manipulations are not possible when using an orphan mesh, an approach of model generation and material property assignment that allows geometric alteration is required.

Implants used for the treatment of fractures need to satisfy three key clinical requirements and consequent mechanical demands arising from them: they must support fracture healing; they must not fail during the healing period; and they should not loosen or cause patient discomfort [20]. The corresponding mechanical demands are interfragmentary motion, plate stresses, and bone strains at the bone‐screw interface [21]. Examination of these variables in subject‐specific models will lead to knowledge gains in the field that could improve future treatment approaches and implant design. This study aims to achieve two objectives: first, to develop a model‐generation approach that enables the manipulation of bone geometry and application of locking plate treatment; and second, to use these models to investigate how subject−specificity influences locking‐plate treatment for distal femur fractures by examining the mechanical demands placed on them over the course of healing.

## 2 Methods

2.1

Three CT scan datasets of cadaveric legs were used in this study (ethics statement related to these scans is provided at the end of this article). An Aquilion ONE CT scanner (Toshiba Medical Systems Ltd., Crawley, UK) was used. The scans captured the legs with a resolution of 35 μm isotropic voxels, with each slice measuring 0.5 mm. The table had a pad containing hydroxyapatite calibration phantoms (INTable Callibration Phantom Couch Pad, Image Analysis Inc., Kentucky, USA). These phantoms had the densities 0, 75, and 150 mg/cm^3^. The data was exported in the DICOM format.

The femoral geometry for each of the selected scans (henceforth Subjects A, B, and C) was generated using CT image segmentation within Simpleware ScanIP [Synopsys, Exeter, UK]. The segmented femur voxel group was infilled and smoothened using a Gaussian filter with a sigma of one pixel before being converted to a tessellated surface mesh. The surface mesh was then converted to a solid part in Autodesk Fusion (Autodesk, San Francisco USA). A FE model from the digital scan of a large fourth‐generation Sawbones Synthetic Femur (Product 3406, Sawbones, Vashon, USA) was included as the fourth subject in the study.

**FIGURE 1:**
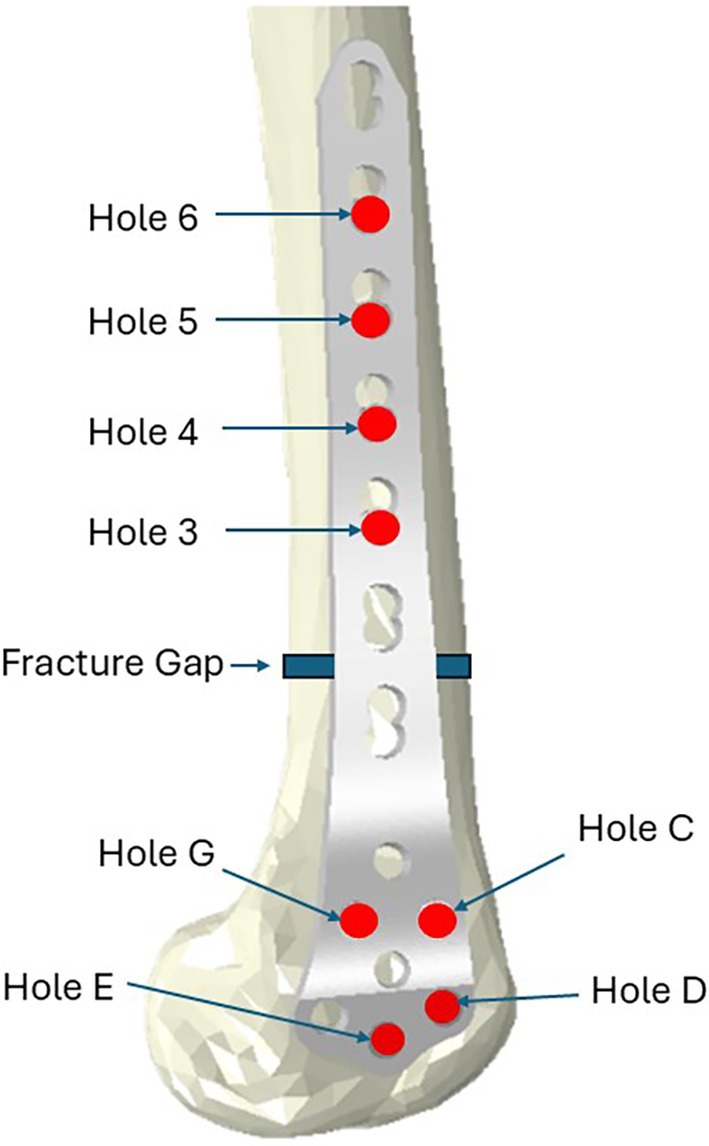
Distal femur fracture and treatment approach simulated.

For the distal femur fracture study, a simplified 7‐hole locking plate geometry (without screw threads or surface bevels) was imported to the CAD space for all femur models and aligned to the bone with an attempt to follow surgical guidance on simple extraarticular distal femur fractures [22]. Placement details are provided in Table 1. The same screw arrangement along with the same plate was employed for all subjects, with holes C, D, G, E, and 3–6 filled as shown in Figure 1. The distance between the bone and plate varied between 1.9 and 2.4 mm across the four bone models considered. Material was removed from the bone parts on overlap with the screw parts, creating zero‐interference screw‐holes.

**TABLE 1:** Measurements of plate placement.

Subject	Plate distal clearance (mm)	Plate anterior clearance (mm)	Sagittal plane alignment (°)	Bone‐plate distance (mm)
A	15.5	13.6	23.6	2.1
B	16.1	13.8	16.0	1.9
C	16.4	13.5	19.0	2.3
Sawbones	9.9	15.0	21.5	2.4

**FIGURE 2:**
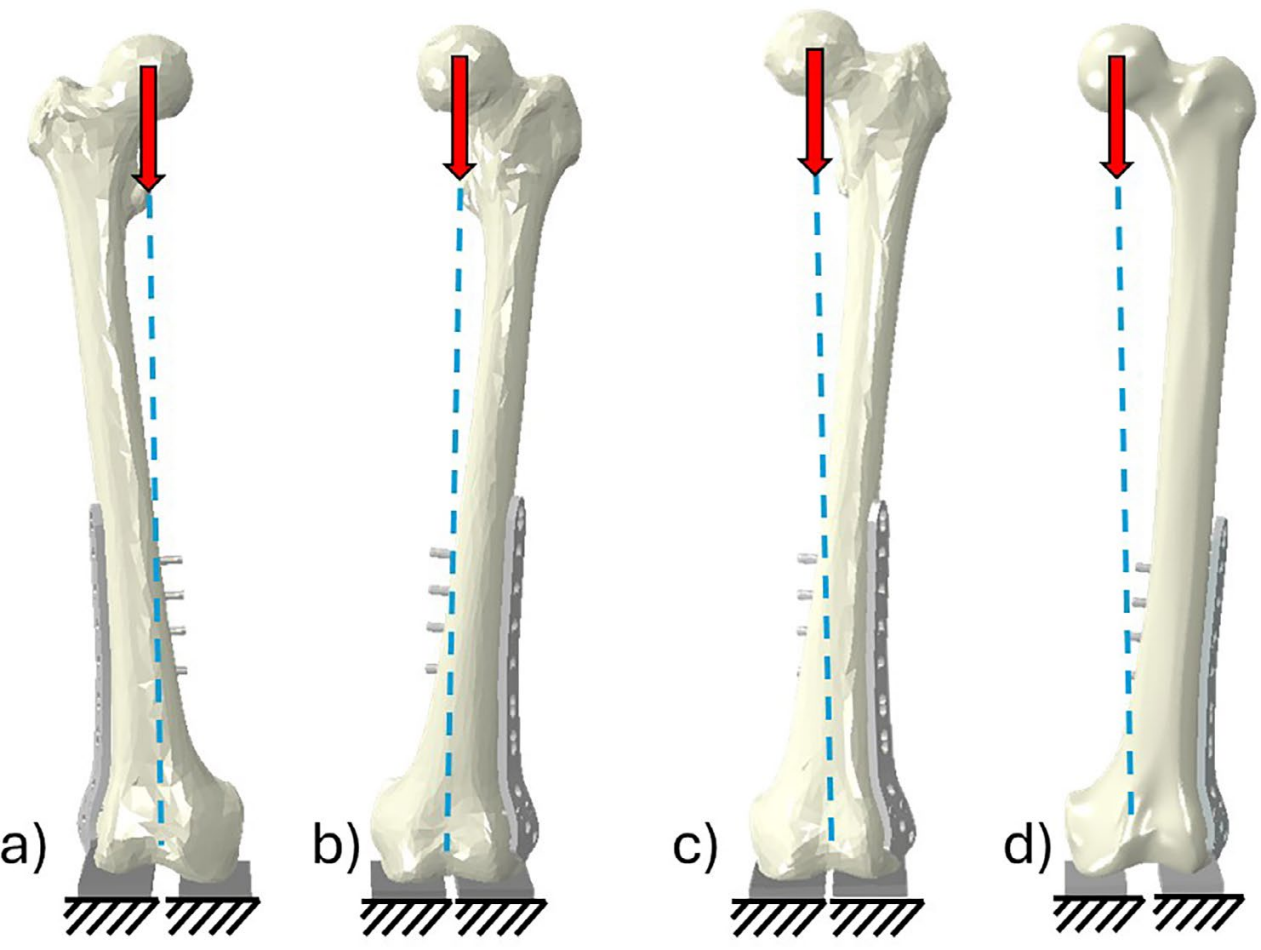
Boundary and loading conditions applied to each model. (a) Subject A, (b) Subject B, (c) Subject C, (d) Sawbones.

The completed osteosynthesis assemblies were imported to Abaqus (Dassault Systèmes, Vélizy‐Villacoublay, France) for FE analyses. A simplified in vitro single‐leg stance loading arrangement was used (Figure 1), in which a load was distributed through the femoral head, acting along a mechanical axis from the center of the femoral head to the center of the condylar contact surface, shown in Figure 2. The condyles were supported on flexible pad parts (*E* = 25 MPa) with fixed bases and a tied constraint between the bone and pad. The femoral head was restrained to allow motion only in the frontal plane. Our analysis of this loading arrangement applied to intact femur models (without plating) for each subject found the balance of reaction force in the medial compartment to be between 54% and 57% of the applied load, matching experimentally derived ranges [23]. The applied load was selected as 238% body weight of a 70 kg individual, corresponding to the peak force during gait [24].

As the aim of the study was to compare different subject‐specific models, a simplified analysis was conducted; the screw‐bone and screw‐plate interactions were both tied. Previous research has shown that while this approach has negligible influence on interfragmentary motions [25], it does not accurately characterize the strain environment around the screws. However, this study assumed that this approach would be satisfactory for comparison among subject‐specific models.

Each complete osteosynthesis model was meshed with linear tetrahedral elements, with an average element edge length of 1 mm, which is much smaller than the 3 mm minimum for modelling long bones previously established [26]. This mesh density was required to ensure appropriate assignment of subject‐specific material properties (discussed later). This meshing resulted in between 2,500,000 and 3,000,000 elements used to mesh each subject femur, 164,759 elements used to model the plate, and between 8600 and 8900 elements to model each screw, dependent on their length.

Medical grade stainless steel, 316 L, with *E* = 200 ± 20 GPa and high ductility is the most commonly used material for bone plates and a range of values of Young's modulus within this range have been previously used. In this study the plate and screws were assigned Young's modulus, *E* = 193 GPa, and Poisson's ratio, *ν* = 0.3. Young's moduli for bone were assigned in Abaqus using a field variable to enable representation of heterogeneity using the process described below. A constant Poisson's ratio of 0.3 was used for bone.

A node‐wise mapping method was used to assign material properties to the bone continuum. The raw CT data for each femur was processed using a bespoke MATLAB code to produce a database for each subject specifying the spatial limits of the scan space, the voxel spacing in each direction, and the full set of CT data expressed as radiological attenuation in Hounsfield Units (HU). The database file for each subject was input to the FE analysis in Abaqus using user subroutine UEXTERNALDB and applied as a temperature field to the bone geometry using UTEMP. This subroutine used the coordinates of each node to find the closest voxel to that node, and used the HU of that voxel to assign a Young's modulus. This method is discussed later in detail.

Numerous studies report relationships between radiological attenuation and Young's modulus of bone. This study employed the relationship given by Cong et al. [27] to determine the ash density of bone, $\rho_{\mathrm{ash}}$, from HU as:

(1) $$\rho_{\mathrm{ash}}=-0.009+0.0007\;\mathrm{HU}$$

followed by relationship suggested by Schileo et al. [28] to evaluate apparent density as

(2) $$\rho_{\mathrm{app}}=\rho_{\mathrm{ash}}/0.6$$

and finally, Young's modulus using a relationship for the femur proposed by Morgan et al. [29]:

(3) $$E=6950\times \rho_{\mathrm{app}}^{1.49}$$

The material properties assigned to the Sawbones model were 150 MPa for trabecular bone and 18 GPa for cortical bone [30]. While prior research has shown that the microarchitecture of trabecular and cortical bone yields different relationships between HU and stiffness, Schileo et al. [28] found significant agreement between experimental results and FE models using a single material formulation. The same approach has been used in this study. It is important to note that both cortical and trabecular bones are regarded as orthotropic [31, 32] and some studies have employed these properties in the evaluation of fracture treatments [16, 33, 34]. A simpler homogeneous transversely isotropic model has also been previously considered for Sawbones [35]. Currently, there are no agreed approaches to assign anisotropic material properties to subject‐specific models that are typically constructed from scans, which provide heterogeneous properties based on HU with no directional information.

A previous study suggested that the interfragmentary fracture gap, which remains after operative reduction, should ideally be minimal but not larger than 2–4 mm [36]. In this study, a fracture gap of 4 mm was applied in each subject‐specific model. To simulate different stages of healing, the gap region was filled with material with low Young's modulus. The material in the fracture gap was chosen to be indicative of healing callus at 3 stages: 1 MPa (granulation tissue), 10 MPa (fibrocartilage), and 100 MPa (woven bone) [37]. These stages will henceforth be referred to as C1, C2, and C3 respectively. While the accurate modelling of mechano‐regulated healing using Finite Elements has seen improvement in recent years [38], this study takes a more representative approach to approximate healing to enable focus on the influence of subject‐specificity.

**FIGURE 3:**
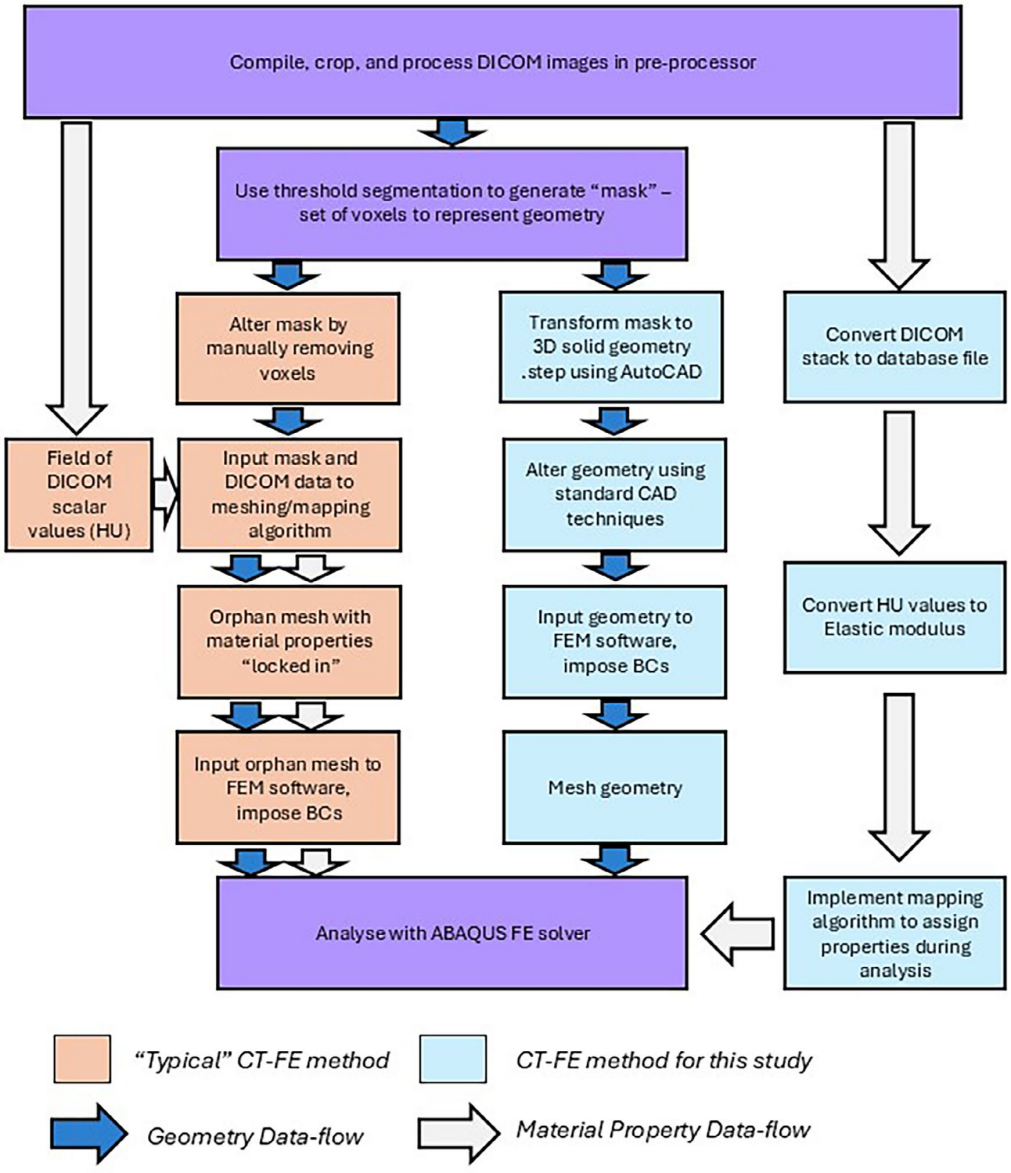
Comparison of typical the conventional CT scan to FE model workflow to the method proposed in this study.

The nodewise method of assigning material properties to the bone continuum [39] is not generally preferred, as the element stiffness resulting from the interpolation evenly weights the contributions from each node, irrespective of the volume contribution of each parent voxel to the element volume [40]. As a result, a volumetric averaging approach is more often used, which applies material stiffness to the element directly, rather than to each node [41]. However, with increasingly small elements, the error imposed by this method is likely to be minimal. The benefit of using this method is that the handling of geometric and material property datasets can be separated till the point of analysis. Figure 3 compares the approach adopted in this study that readily permits manipulation of the geometry to model complex osteosynthesis with the traditional approach which does not.

Analysis of the four FE models (subject‐specific models A, B, C and Sawbones) was conducted in Abaqus Standard using linear static analyses. Each model was analyzed once with each of the healing callus properties to assess the osteosynthesis construct performance across a range of healing times.

The osteosynthesis construct performance was assessed over the following metrics:

- Interfragmentary motion normal to the fracture gap at both the near and far cortex, and transverse to the fracture gap at the far cortex only.
- Von Mises stress in the plate, in the active bridging region where the highest stresses are expected.
- Equivalent Strain Volume (EqSV) of bone at the screw interface, taken as the volume with tensile principal strain over 0.5% or compressive principal strain over 0.7%.

Variation of material properties in subject‐specific models was examined. The average values of cortical and trabecular bone stiffness in each specimen were also evaluated.

Models in the verification study were compared using the femoral head deflection and the maximum predicted von Mises stress in cross‐sections of the bone at the proximal metaphysis, mid‐diaphysis, and distal metaphysis (20%, 50%, and 80% of the femur length).

## 3 Results

### 3.1 Verification of Material Mapping Approach

A brief study was conducted to compare the proposed method of material property assignment to the standard method available in the commercial package ScanIP, which uses a volumetric averaging approach. We used an intact femur model for this purpose as it is not possible to manipulate the geometry (for creating fracture, screw holes etc.) after material assignment and meshing in ScanIP. A model of Subject C was generated using the proposed method and by using the CT‐FE functions within ScanIP. The default bone material property definition in ScanIP was used for this comparison in which the applied Young's Modulus, *E* (MPa) is given by

(4) $$\rho =1.31\times 10^{-10}+1.067\times 10^{-12}\times \mathrm{HU}$$

(5) $$E=-331+4.56\times 10^{12}\rho$$

where HU is the raw attenuation in Hounsfield units and *ρ* is the mineral density of the bone tissue. The range of material properties calculated by ScanIP was divided into 8 distinct bins, and each element was assigned to a bin based on the volume‐averaged radiological attenuation value within the element. The resulting Young's modulus values assigned to each element correspond to the midpoint of a given bin. This preparation in ScanIP resulted in an orphan mesh model comprised of 455,307 linear interpolation tetrahedral elements with material properties assigned as described above.

Two models of Subject C were generated using the node‐wise material property assignment method described earlier. One of these models used a mesh of similar density to the model produced using ScanIP (454,916 tetrahedral elements total), and the other model used a significantly denser mesh (2,840,843 tetrahedral elements total and with an average edge length of around 1 mm). The loading and boundary conditions applied to each model followed the schematic provided in Figure 2, with the condylar surface restrained and a single load applied to the femoral head acting along the biomechanical axis. Neither the plate nor fracture gap was applied to the models as the aim of this computation was to compare the efficacy of two methods of modeling the bone continuum.

**FIGURE 4:**
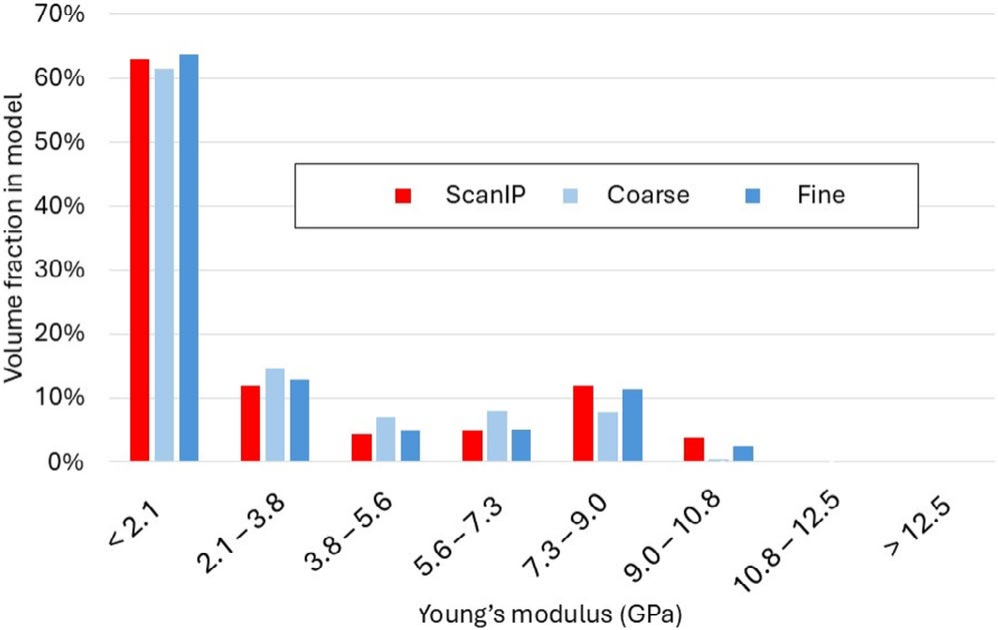
Comparison of Young's modulus distribution achieved in bone modeled with ScanIP with the proposed new method (with a coarse and fine mesh). Here the moduli are computed using Equations 4 and 5.

The volume fraction of elements within specific ranges of material properties was calculated for each of the three models described above—one with material properties assigned by ScanIP and two using the proposed approach with different mesh densities. The limits of these ranges were determined by the piece‐wise “bin” assignment of material properties in ScanIP. A histogram of material property volume fractions (based on Equations 4 and 5) is shown in Figure 4.

The total volume fractions of elements with low Young's modulus (< 2.1 GPa) were found to be around 60% for all three models, representing both trabecular bone and material within the medullary cavity. The volume fraction of elements in higher stiffness bins was also relatively consistent amongst subjects, with the higher density mesh showing better agreement with the ScanIP model. Notably, only the ScanIP model had some elements in the highest bin of material properties (> 12.5 GPa).

**FIGURE 5:**
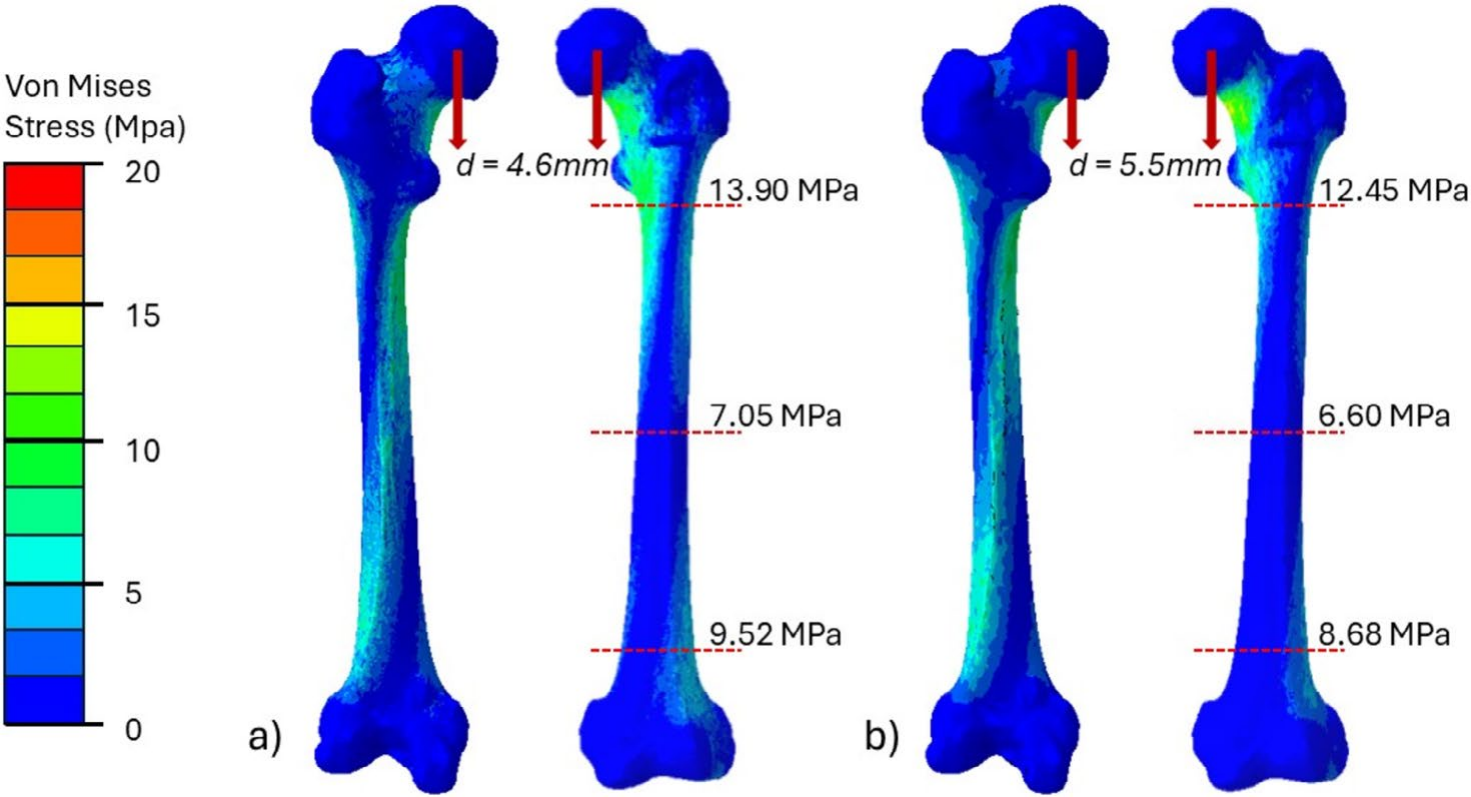
Comparison of the femoral head deflection and peak stresses at key anatomical locations in the verification models developed with (a) ScanIP and (b) the proposed approach.

Our subsequent analyses with locking plates used finer meshes to represent all components satisfactorily. It can also be seen that the finer mesh provided a material property distribution that was similar to the volume‐averaged model. Figure 5 compares results of femoral head deflection and von Mises stress for the fine mesh and the ScanIP mesh. The net deflection of the femoral head (*d*) was calculated and resolved in the direction of load application. The ScanIP model (ScanIP) resulted in a lower total tip deflection (*d =* 4.6 mm), indicating that this model has a higher stiffness on average. Our significantly finer mesh expectedly provided a larger deflection of 5.5 mm. The von Mises stress contours are visually similar though high stresses generally seem more diffused in the ScanIP model. Figure 5 also provides peak von Mises stress values calculated at the three cross‐sections. These are not single element peaks but averaged over a 3 mm diameter region. These results show higher maximum stresses in the cortex of the ScanIP model, indicating that jumps in element stiffness caused by piecewise assignment may create stress risers.

### 3.2 Subject‐Specific Bone Stiffness Characterization

**FIGURE 6:**
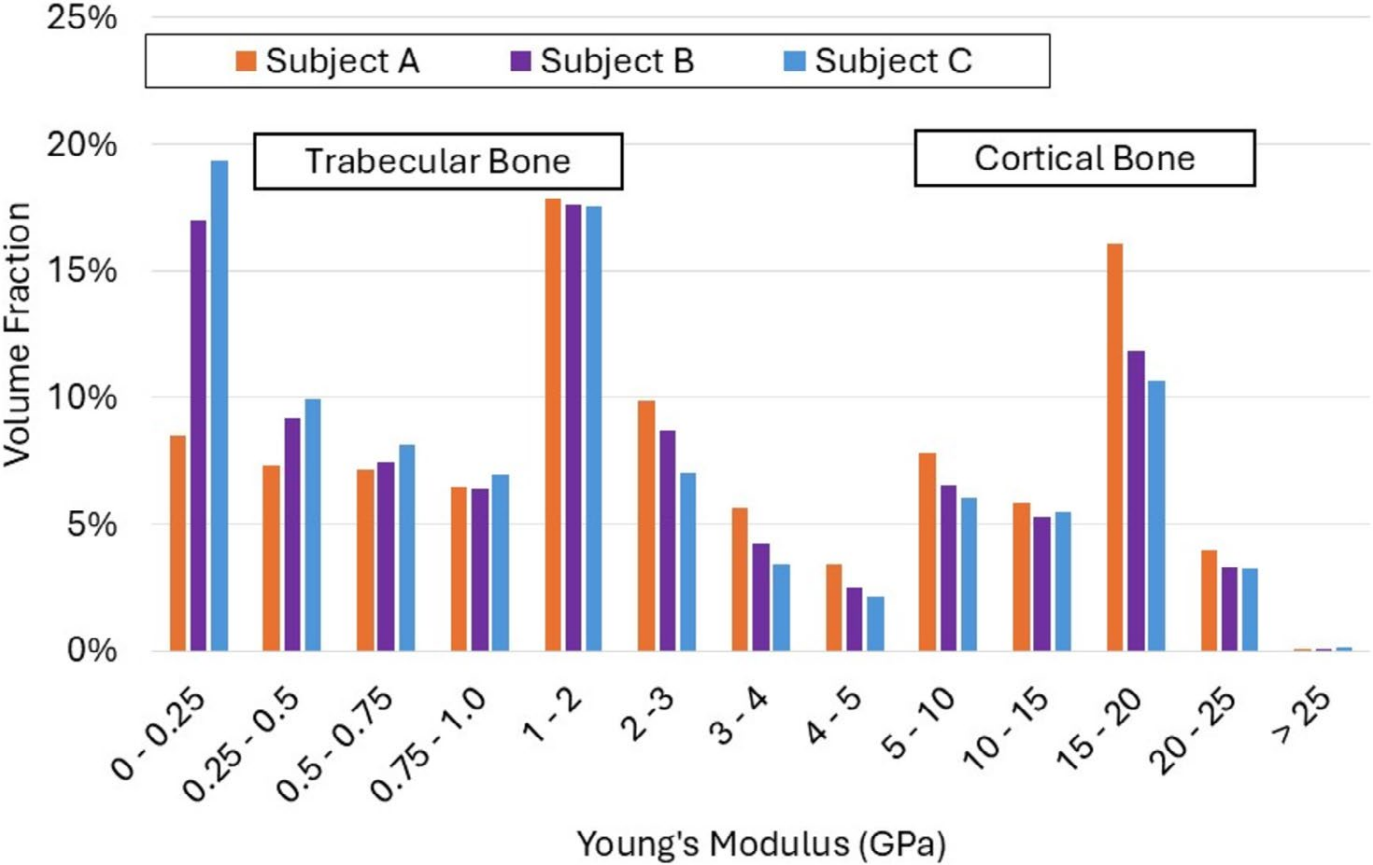
Comparison of Young's moduli distribution in subjects A, B, and C. when implementing the proposed approach. The moduli are computed using (Equations (1), (2), (3)).

The distribution of element stiffness by volume in the three subject‐specific models is shown in Figure 6. The average stiffnesses of cortical and trabecular bone determined by element sampling in each model are presented in Table 2. The Young's moduli characterization is henceforth based on Equations ((1), (2), (3)).

**TABLE 2:** Average Young's modulus computed for cortical and trabecular bone for each subject and in the Sawbones two‐phase model.

Model	Cortical bone (GPa)	Trabecular bone (GPa)
Subject A	17.69	0.62
Subject B	17.00	1.05
Subject C	16.20	0.69
Sawbones	18.00	0.15

The histogram in Figure 6 shows clear differences in stiffness characteristics between the three subjects studied. Subject A consistently showed the lowest volume fraction of low‐stiffness elements (in the 0–1 GPa range), but the highest proportion of high‐stiffness elements (from 1 to 25 GPa). By contrast, Subject C had higher proportions of low‐stiffness elements and lower proportions of high‐stiffness elements. Subject B typically fell somewhere between the other two subjects. This pattern provides insight into the general differences in bone quality between the subjects. This distribution cannot be readily compared with the Sawbones model. For trabecular bone, however, average Young's moduli obtained for subject‐specific models were higher than those for the Sawbones model (0.15 GPa). The highest difference was observed in Subject B, which had an average trabecular stiffness of 1.1 GPa.

This interpretation is substantiated by the results presented in Table 2. Subject A had the highest average stiffness of cortical bone at 17.7 GPa, and subject C had the lowest cortical stiffness at 16.2 GPa.

### 3.3 Interfragmentary Motions

**FIGURE 7:**
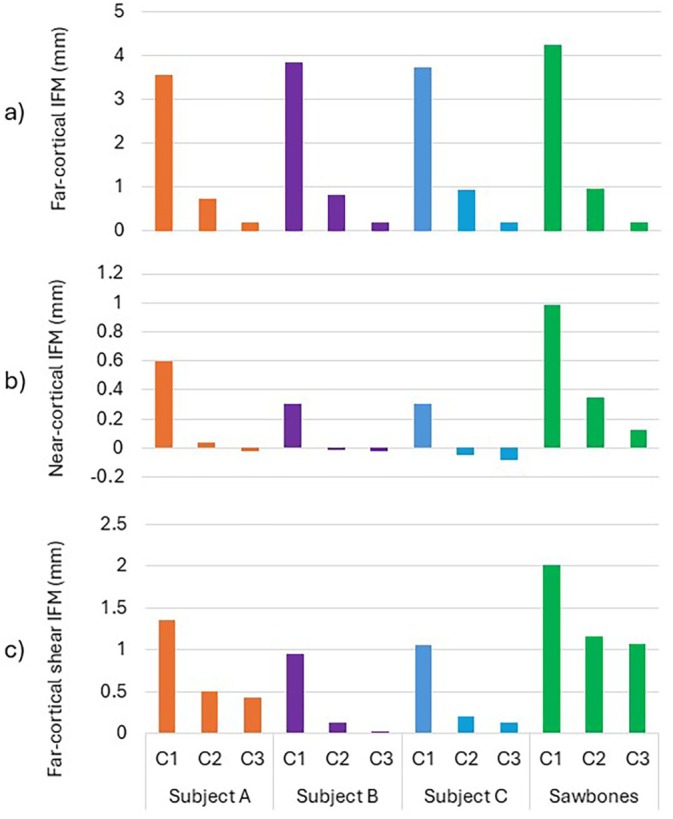
Comparison of (a) axial IFM at the far cortex, (b) axial IFM at the near cortex, and (c) shearing IFM at the far cortex for the four models considered. C1, C2 and C3 are the three stages of healing representing granulation tissue, fibrocartilage, and woven bone in the fracture gap respectively.

All subsequent analyses were with the fracture induced and locking plate applied to all three subject‐specific models and to the Sawbones model with material assignments using the proposed mapping approach. The interfragmentary motions (IFM) were computed in each case for the peak load considered. The largest IFM was found to be in the fracture gap at the far cortex (furthest away from the plate), and the smallest at the near cortex. As shown in Figure 7a, the pattern of far‐cortical IFM was similar in all models at all three stages of healing considered, with IFM decreasing dramatically as healing progressed. The Sawbones model exhibited the highest IFM at all healing stages. The largest difference between the Sawbones model and a subject‐specific model was observed for Subject A, in which the observed far‐cortical IFM was 16% lower than predicted by the Sawbones model at the earliest stage of healing considered. Differences in IFM calculations became less pronounced when higher stiffnesses were applied to the fracture gap. At stage C3, the observed differences were very small.

The good agreement between the generic model and the subject‐specific models throughout the simulated stages of healing indicates that generic geometry models can predict the magnitude of far‐cortical IFM, as well as probable changes in the IFM pattern over the course of healing. It also shows that subject specificity has relatively little impact on far‐cortical IFM.

By contrast, the axial IFM measured at the cortex nearest to the plate was not similar between models. Figure 7b shows the trends of near‐cortical axial IFM for each model at each stage of healing. The generic Sawbones‐based model exhibited the highest IFM at 1 mm compared to 0.6 mm for Subject A and 0.3 mm for subjects B and C in the first stage of simulated healing.

Figure 7c shows IFM in shear at the far cortex, which arise due to asymmetry of geometry and loading. The highest shear IFM was around 2 mm in the Sawbones model, which is 103% higher than the shear IFM of 0.96 mm for Subject B. This is similar to the comparison between the same two models in near‐cortical axial IFM, where the relative difference (0.98 mm and 0.31 mm) was over 300%.

### 3.4 Plate Stresses

In all of the models at the earliest stage of healing, the peak von Mises stress observed in the locking plate occurred on the medial aspect of the plate, at the screw‐hole nearest to the fracture gap. This pattern is consistent with combined axial compression and minor axis bending through the plate.

However, at higher stages of healing, callus in the fibrocartilage and woven bone stages exhibited the peak plate stresses on the posterior aspect of the plate, indicating that once healing has passed a certain threshold of load‐sharing through the callus, the plate acts predominantly in major‐axis bending.

**FIGURE 8:**
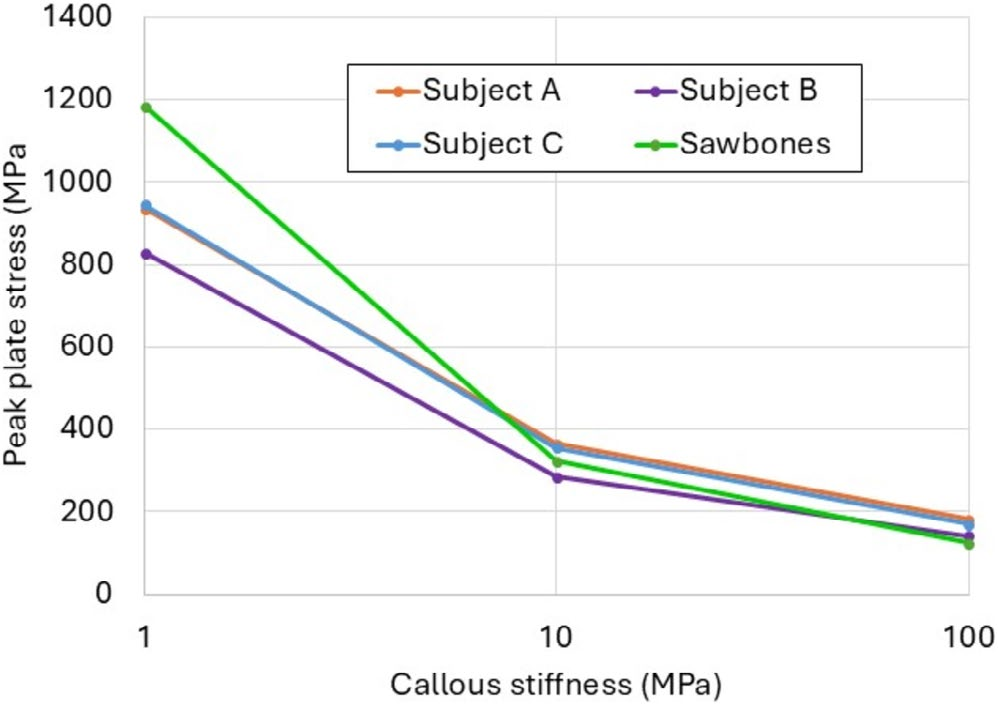
Peak plate stress computed in each model as healing progresses.

Figure 8 shows the development of peak plate stress against callus stiffness for all models. In the first instance of callus healing, the Sawbones model predicted a much a higher peak stress (1183 MPa) in comparison to the subject‐specific models, with a maximum error of 30% compared to Subject B (827 MPa). Inter‐model variations decreased significantly at later stages of healing. In healing stage C3, the greatest difference was between the sawbones model (123.8 MPa) and Subject A (181.3 MPa).

### 3.5 Strains Around Screw Insertion Sites

Bone yielding and failure have been known to be based on strain rather than stress [32, 42, 43]. Therefore, we computed the strain distribution around the screw insertion sites comprising compressive strains in the direction of primary screw forcing and tensile strains developed in the hoop direction and “behind” each screw in the forcing radial direction due to tied constraints. These were regarded as a measure of performance as screw loosening is a common failure mode of locking plates and is caused by high strains in the bone due to force transfer from the screws [16].

**FIGURE 9:**
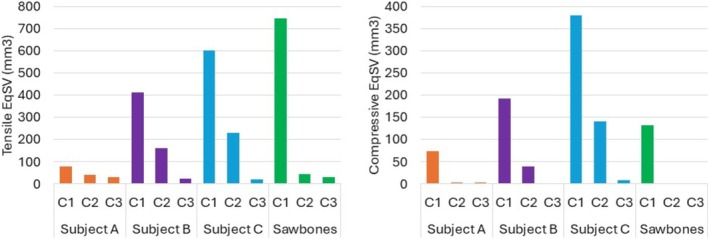
Comparison of volume of highly strained bone (EqSV) exceeding assigned thresholds in (a) tension and (b) compression.

The volume of bone around the screws with maximum principal (tensile) strain exceeding 0.5% and or minimum principal (compressive) strain below −0.7% was computed and is shown in Figure 9. These values are based on typical yield strain values of bone [33, 34].

For all models considered, EqSV (bone volume above threshold strain) was higher for tensile strains than compressive strains, likely because the compressive threshold of bone is higher than the tensile threshold and also since the tied screw–bone interface was modeled. Strains in the bone were generally higher in the distal femur than in the diaphysis.

Notable variation in EqSV was observed between models, as shown in Figure 9. The lowest EqSV occurred in Subject A for both tension and compression while the highest was for subject C. In the subject‐specific models, high tensile EqSV was accompanied by high compressive EqSV. This pattern was not borne out by the Sawbones model, which showed the highest tensile EqSV out of all the models but only middle‐range compressive EqSV. This pattern indicates a significant difference in the way that screw‐bone interfaces behave when using generic Sawbones‐style models compared to subject‐specific models. This observed difference is clearly related to the significant difference in bone stiffness variation in subject‐specific models and in the sawbones model. As previously noted, while the bone stiffness calculated by the subject‐specific models in this study is consistent with values of Young's moduli found in the literature, the bipolar stiffness distribution in the Sawbones model does not represent reality. Low trabecular bone stiffness creates artificial flexibility in the calculated behavior of uni‐cortical screws used in the distal femur, which are primarily anchored in trabecular bone.

## 4 Discussion

The study introduces a novel approach to assess how subject‐specific factors influence the effectiveness of the same treatment option. The proposed approach can be applied to evaluate how different patients will respond to a given implant or how a single patient will respond to different implants.

The proposed method of mapping the elastic moduli of bone onto the manipulated geometric part was successful. In our verification experiment, there was a 20% difference in femoral head deflection calculated when using our bespoke node‐averaged mapping approach compared to the commercial volume‐averaged mapping approach. This result agrees with Taddei et al. [44] who found that node‐averaged mapping provides good predictions as the mesh is refined. The method in this study allowed for the bone geometry to be arbitrarily manipulated without creating additional work later in the modelling process. This is considered preferable to similar methods such as those used by Aquilina at al. [18] which require fracture gap material to be removed voxel by voxel during CT image segmentation and do not allow more complex geometry like screw holes to be introduced.

This study also provides valuable insights on output parameters that are sensitive to subject‐specificity and those that are less so in the treatment of distal femur fractures using locking plates. Mechanical demands such as plate stress and interfragmentary motion at the far cortex were not significantly affected by subject‐specificity and the generic Sawbones‐based model, despite notable differences in overall geometry and elasticity distribution. The largest differences between models were observed when comparing the strain‐state in the bone stock surrounding screw insertion sites, where calculations of volume of highly strained bone in subject‐specific models varied by over 600% and variations between the generic model and subject‐specific models were up to 700%. We did not incorporate screw threads and used tied constraints instead of friction at the bone‐screw interface. Previous studies have shown that threads can cause strain concentrations, though these predominantly arise in dynamic compression plates in comparison to locking plates [4]. Strain environment in the bone is also influenced by use of tied constraints, though it does not affect interfragmentary motions [25]. MacLeod et al. [25] showed that the frictional and tied representations did not have significantly different peak tensile and compressive strain values (the frictional interface had higher peak compressive strains while the tied interface had higher tensile strains). The focus of the current study was to examine the trends and check whether interfacial strains were influenced by patient‐specificity. We found that they were and that patient specificity would be an influence even if the constraint conditions were made frictional.

The finding that computation of plate stress and far‐cortical IFM are less sensitive to bone quality while strains in the bone at the bone‐screw interface agree well with previous research on the subject. Previous studies on tibial midshaft fractures treated with unilateral fixators [33] and Ilizarov ring fixators [34] considered bone properties varying with age and found that while bone volume undergoing inelastic strain at the pin or wire interface significantly increased as bone quality decreased, the IFM remained largely unaffected. A similar conclusion was reached by MacLeod et al. [16] who considered tibial midshaft fractures treated with locking plates. These previous studies used orthotropic properties that varied across the cross‐section and, therefore, were only partially heterogeneous. The current study shows that with fully heterogeneous properties, local strains can vary significantly. Additionally, the observation in our study that differences in plate stress and far‐cortical IFM between subject models reduced in later stages of healing indicates that the impact of subject‐specific variations reduces over time.

There are no previously published results indicating the level of variation found here between calculations of near‐cortical IFM and shear IFM. This variation is a result of a range of factors. Due to differences in the geometry of each model, assembly characteristics such as the distance between the plate and the bone surface were not uniform. Another possible explanation for this pattern is the difference in average trabecular bone stiffness in each model. Previous studies [16, 33, 34] used screws, pins, or wires that traversed the entire bone shaft, and the loads experienced were relatively axial in comparison to this study. A lower stiffness of trabecular bone stock would allow increased screw motion within the bone, resulting in higher dynamism of the plate assembly. The variation in shear IFM is more likely to be caused by the individual geometry of the subjects resulting in different torsional motions at the far cortex.

We noted that while far‐cortical IFM is largely generated by plate bending, near‐cortical axial IFM is strongly influenced by a combination of axial compression, bending of the plate, and bone‐screw interaction. The dominance of bending is further illustrated in the later stages of healing, where the near‐cortical IFM indicates tensile straining in some cases. Shear IFM is influenced by both subject‐specific geometry and material property distribution. Locking plates such as the one modeled in this study are designed to reduce the toggling dynamics between the screws and the plate; however, toggling may still occur between the screws and the bone [4, 45] though it has not been modeled in this study. This variation indicates that minor differences in subject bone quality and geometry may have a significant impact on these measures of IFM and thus on healing outcomes.

The levels of peak plate stress calculated by the models used in this study are high, particularly in the early stages of healing. For consistency, we applied identical loading of 238% body weight for a 70 kg individual in all cases. Patients are unlikely to bear full weight in the early stages of healing. Localized stress concentrations around the screw‐holes of the plate, as seen in this study, have been documented extensively in the literature [46, 47]. Yield stress of surgical‐grade steel has been reported to be substantially lower [48] than the peak stresses predicted by the models in this study. This indicates that some localized irreversible plate deformations are likely in the early stages of healing if the patient weight‐bears fully. Previous simulations have also predicted high values of stresses in the locking plates used in long bone fractures [49]. However, the aim of this study was not to predict the true physical state in a clinical application of locking plate fixation of distal femoral fractures, but rather to examine the significance of subject specificity. The trends that emerge from this study are comparable to several previous studies.

To the best of our knowledge, this is the first study that evaluates the influence of subject specificity for similar fracture and treatment. However, it is important to recognise that, in addition to heterogenous material properties being different, the overall geometry of subjects is also different. Consequently, the application of locking plates cannot be completely identical, as discussed earlier. The variation of geometry and treatment will also result in varying displacement of the femoral head in all directions, which in this study was restrained to stay in the frontal plane.

## 5 Conclusions

The study shows that the proposed method, which enables manipulating bone geometry while retaining subject‐specific properties, permits the creation of models of different subjects with similar fractures receiving similar treatments. This novel approach, which enables manipulating bone geometry while retaining subject‐specific properties, can be used to evaluate the influence of subject‐specificity for other orthopaedic simulations. The study shows that while some output parameters, such as far‐cortical IFM, are less sensitive to subject‐specificity, others, such as bone strains at the bone‐screw interface, are very sensitive. The effect of subject‐specificity reduces as healing progresses. The study also shows that for problems such as this, the two‐material Sawbones model is less likely to reflect reality.

## Ethics Statement

This statement relates to the subject‐specific CT scans used in this study. This investigation took place at the Department of Anatomy, University of Edinburgh. The experiment was carried out in accordance with the Human Tissue (Scotland) Act 2006. The imaging was undertaken under the supervision of a licensed anatomist at the Clinical Research Imaging Centre, Queens Medical Research Institute, Royal Infirmary of Edinburgh, University of Edinburgh.

## Conflicts of Interest

The authors declare no conflicts of interest.

## Data Availability

The data that support the findings of this study are available on request from the corresponding author. The data are not publicly available due to privacy or ethical restrictions.
